# Airway pressure release ventilation during acute hypoxemic respiratory failure: a systematic review and meta-analysis of randomized controlled trials

**DOI:** 10.1186/s13613-019-0518-7

**Published:** 2019-04-04

**Authors:** Andrea Carsetti, Elisa Damiani, Roberta Domizi, Claudia Scorcella, Simona Pantanetti, Stefano Falcetta, Abele Donati, Erica Adrario

**Affiliations:** 1 Anesthesia and Intensive Care Unit, Azienda Ospedaliero Universitaria Ospedali Riuniti, Ancona, Italy; 2 Anesthesia and Intensive Care Unit, Università Politecnica delle Marche, Ancona, Italy

**Keywords:** Airway pressure release ventilation, Acute respiratory failure, Acute respiratory distress syndrome, Meta-analysis

## Abstract

**Background:**

Airway pressure release ventilation (APRV) has been considered a tempting mode of ventilation during acute respiratory failure within the concept of open lung ventilation. We performed a systematic review and meta-analysis to verify whether adult patients with hypoxemic respiratory failure have a higher number of ventilator-free days at day 28 when ventilated in APRV compared to conventional ventilation strategy. Secondary outcomes were difference in PaO_2_/FiO_2_ at day 3, ICU length of stay (LOS), ICU and hospital mortality, mean arterial pressure (MAP), risk of barotrauma and level of sedation. We searched MEDLINE, Scopus and Cochrane Central Register of Controlled Trials database until December 2018.

**Results:**

We considered five RCTs for the analysis enrolling a total of 330 patients. For ventilatory-free day at day 28, the overall mean difference (MD) between APRV and conventional ventilation was 6.04 days (95%CI 2.12, 9.96, *p *= 0.003; *I*^2^ = 65%, *p *= 0.02). Patients treated with APRV had a lower ICU LOS than patients treated with conventional ventilation (MD 3.94 days [95%CI 1.44, 6.45, *p *= 0.002; *I*^2^ = 37%, *p *= 0.19]) and a lower hospital mortality (RD 0.16 [95%CI 0.02, 0.29, *p *= 0.03; *I*^2^ = 0, *p *= 0.5]). PaO_2_/FiO_2_ at day 3 was not different between the two groups (MD 40.48 mmHg [95%CI − 25.78, 106.73, *p *= 0.23; *I*^2^ = 92%, *p *< 0.001]). MAP was significantly higher during APRV (MD 5 mmHg [95%CI 1.43, 8.58, *p *= 0.006; *I*^2^ = 0%, *p *= 0.92]). Then, there was no difference regarding the onset of pneumothorax under the two ventilation strategies (RR 1.94 [95%CI 0.54, 6.94, *p *= 0.31; *I*^2^ = 0%, *p *= 0.74]). ICU mortality and sedation level were not included into quantitative analysis.

**Conclusion:**

This study showed a higher number of ventilator-free days at 28 day and a lower hospital mortality in acute hypoxemic patients treated with APRV than conventional ventilation, without any negative hemodynamic impact or higher risk of barotrauma. However, these results need to be interpreted with caution because of the low-quality evidence supporting them and the moderate heterogeneity found. Other well-designed RCTs need to be conducted to confirm our findings.

## Introduction

Acute hypoxemic respiratory failure is a common reason for patients to be admitted to the intensive care unit (ICU). An international study showed an incidence of acute respiratory distress syndrome (ARDS) of 10.4% in ICU critically ill patients with an hospital mortality reaching 46.1% for most severe cases [1].

A protective ventilation strategy using low tidal volume (LTV) and a plateau pressure lower than 30 cmH_2_O is widely accepted to limit ventilator-induced lung injury [2], and it currently represents the intervention able to reduce mortality supported by the strongest evidences [3].

Airway pressure release ventilation (APRV) was described for the first time by Stock and Downs [4] and consists in a time-triggered, pressure-limited and time-cycled ventilation mode in which the pressure was alternated from a high level (*P*_high_) applied for a prolonged time (*T*_high_) to maintain adequate lung volume and alveolar recruitment, to a low level (*P*_low_) for a short period of time (*T*_low_) where most of ventilation and CO_2_ removal occurs. In contrast to pressure-controlled inverse-ratio ventilation, APRV uses a release valve that allows spontaneous breathing during any phase of respiratory cycle. The rationale behind this approach is to maintain a pressure above the closing pressure of recruitable alveoli for a sustained time, limiting the release time to allow CO_2_ removal but avoiding de-recruitment. Another conceptual advantage to APRV over controlled modes is the preservation of spontaneous breathing, which may promote a redistribution of aeration to the dependent lung regions, less need for neuromuscular blockade and sedation, improved venous return and a better ventilation/perfusion (*V*/*Q*) matching. For this reason, APRV has been considered a tempting mode of ventilation during acute respiratory failure within the concept of open lung ventilation. However, the benefits of APRV over conventional ventilation need to be verified.

The aim of our systematic review and meta-analysis was to verify whether adult patients with hypoxemic respiratory failure have a higher number of ventilator-free days when ventilated in APRV compared to conventional ventilation strategy.

## Methods

The methods and reporting of the systematic review followed Preferred Reporting Items for Systematic Reviews and Meta-analyses (PRISMA) guidelines [5].

### Eligibility criteria

The population of interest included adults (age ≥ 18 years) who were diagnosed with acute hypoxemic respiratory failure (PaO_2_/FiO_2_ < 300 mmHg) and excluded those with severe chronic lung diseases and asthma. The intervention included APRV compared with any type of conventional ventilation. The primary outcome was ventilator-free days at day 28. Secondary outcomes were difference in oxygenation (PaO_2_/FiO_2_ at day 3), ICU length of stay (LOS), ICU and hospital mortality, hemodynamics (mean arterial pressure), risk of barotrauma and level of sedation.

Eligible studies were randomized controlled trials (RCTs). We excluded observational studies, case series and case reports, studies published in abstracts, literature reviews, editorials and studies not conducted in humans. Language was restricted to English.

### Search strategy and data extraction

We searched MEDLINE, Scopus and Cochrane Central Register of Controlled Trials database from their inception to December 2018 for eligible studies. We combined the terms “airway pressure release ventilation,” “APRV,” “acute respiratory distress syndrome,” “ARDS,” “acute lung injury,” “ALI,” “acute respiratory failure.” Results were then filtered for adult human’s studies.

### Study selection and data collection

Two investigators (AC and ED) independently performed the first screen (title and abstract), and the full-text screen of the studies retrieved by our search. The same investigators independently extracted the data. Discrepancies at any step of the process (first screening, full-text screening and data extraction) were resolved by consensus or by the opinion of a third investigator (EA).

### Assessment of risk of bias and quality of evidence

Two trained reviewers (AC and ED) independently assessed the quality of the included studies. We used the Cochrane Collaboration’s tool for assessing risk of bias in RCTs [6]. The included RCTs were assessed for random-sequence generation, allocation sequence concealment, blinding of participants and personnel, blinding of outcome assessment, completeness of outcome data, selective reporting and other sources of bias. Each domain was assessed as low, unclear or high risk of bias. The highest risk of bias for any criteria was used to reflect the overall risk of bias for the study.

### Data synthesis and statistical analysis

The statistical analyses were performed using RevMan, version 5.3 (Cochrane Collaboration). The random-effects model was used for all analyses. Dichotomous variables were analyzed using the Mantel–Haenszel method and were expressed as risk ratio (RR) or risk difference (RD). Continuous variables were analyzed using the inverse variance random-effects model and were expressed as mean differences (MD). For studies that only reported medians, we estimated the mean and standard deviation (SD) using the methods proposed by Wan et al. [7] A two-tailed *p* value of less than 0.05 was set for statistical significance. Heterogeneity was assessed using with the *X*^2^ test and the *I*^2^ test, with *I*^2^ greater than 50% being considered substantial [8]. The possibility of publication bias was assessed by visual estimate of funnel plot and by the regression test of Egger test when 10 or more trials were pooled [6, 9]. As the ventilatory strategy for ARDS patients has been significantly changed after the publication of ARDSNetwork trial [3], a sensitivity analysis for the primary outcome has been performed excluding studies not in line with low tidal volume ventilation.

## Results

### Study selection and study characteristics

**Fig. 1:**
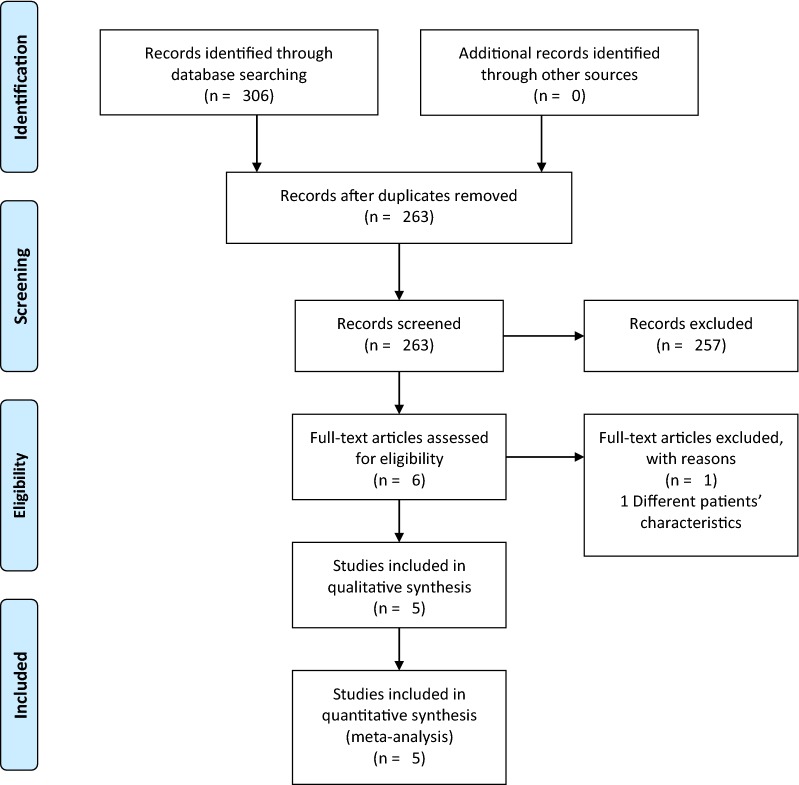
Flowchart of study selection

We identified 306 titles. After removal of duplicates, we screened the titles/abstracts of 263 records and assessed the full text of six articles. Finally, we considered five RCTs for the analysis (Fig. 1) enrolling a total of 330 patients. Table 1 describes the main characteristics of the selected studies. The studies were published from 2001 to 2018. All of them are single-centered RCT. Three studies specifically enrolled only patients with ALI/ARDS including overall 248 patients (75% of the total population considered) [10–12]. One trial specifically included only traumatic patients with acute respiratory failure [13]. Two studies defined ALI/ARDS according to the American-European Consensus Conference on ARDS of 1994 [14], two studies applied the Berlin definition of ARDS [15], and one study did not clearly declare the definition used [12]. One trial compared three ventilation modalities in three groups of patients: APRV, APRV-LTV and volume-controlled LTV (VC-LTV) [16]. For the purpose of our study, we extrapolated data regarding APRV-LTV and VC-LTV.

**Table 1:** Characteristics of the studies included in the meta-analysis

Study	Design	Sample size	Age (years)	Type of patients	PaO_2_/FiO_2_ at baseline (mmHg)	MV before randomization (h)	Intervention	Comparator	APRV initial setting	Primary outcome
Putensen et al. [13]	RCT	30	APRV: 40 ± 19 CV: 42 ± 23	Trauma with acute respiratory failure	250	APRV: 6 ± 3.9 CV: 6 ± 3.9	APRV	PCV	Ph = ↓UIP Vt < 7 ml/kg Pl = LIP + 2 cmH2O Tl = flow 0	Effects on cardiorespiratory function
Varpula et al. [10]	RCT	58	APRV: 50 (38.5–60.5) CV: 44 (35.5–53)	ALI/ARDS P/F < 200 mmHg MV < 72 h	APRV: 150 ± 8 CV: 164 ± 7	39.1 ± 17.5	APRV	SIMV-PC/PS	Ph = ↓UIP Pl = ↑LIP Th = 4 s Tl = 1 s	Ventilator-free days at day 28
Li et al. [12]	RCT	52	APRV: 54.3 ± 8.4 CV: 53.6 ± 9.5	Moderate/severe ARDS	APRV: 119 ± 35 CV: 118 ± 36	Not reported	APRV	SIMV (LTV)	Ph = 30 cmH2O Pl = 0 Th = 4–8 s Tl = 0.4–0.8 s	Unclear
Zhou et al. [11]	RCT	138	APRV: 51.5 ± 15 CV: 52 ± 15.1	ARDS P/F < 250 mmHg MV < 48 h	APRV: 122 ± 47 CV: 138 ± 56	APRV: 24.6 ± 12.6 CV: 22.1 ± 13.5	APRV	LTV	Ph = Pplat Pl = 5 cmH2O Tl > 50% PEFR RR 10–14 bpm MVst = 30% of MVtot	Ventilator-free days at day 28
Hirshberg et al. [16]	RCT	52	APRV: 57 ± 16 APRV-LTV: 57 ± 15 VC-LTV: 51 ± 14	Hypoxemic respiratory failure MV < 24 h	APRV: 109 ± 67 CV: 121 ± 50	Not reported	APRV/APRV-LTV	VC-LTV	Ph = Pplat Pl = 5 cmH2O Th = 4–6 s Tl = 50–75% PEFR	P/F at day 3

*ALI* acute respiratory injury, *APRV* airway pressure release ventilation, *ARDS* acute respiratory distress syndrome, *CV* conventional ventilation, *LIP* lower inflection point, *LTV* low tidal volume ventilation, *MV* mechanical ventilation, *MVst* spontaneous minute ventilation, *MVtot* total minute ventilation, *PEFR* peak expiratory flow rate, *Ph* high level of pressure, *Pl* low level of pressure, *RCT* randomized controlled trial, *RR* respiratory rate, *Th* time for high pressure, *Tl* time for low pressure, *UIP* upper inflection point, *Vt* tidal volume

Three studies clearly enrolled patients within the early phase of respiratory failure, counting less than 24 h of mechanical ventilation before randomization for the majority of patients [11, 13, 16]. Varpula et al. [10] allowed randomization until 72 h from starting of ventilation. Finally, Li et al. [12] did not report the time under ventilator before randomization.

About the initial APRV setting, three studies measured a static pressure–volume (*P*–*V*) curve to identify lower (LIP) and upper (UIP) inflection points and used these data to set pressures [10, 12, 13]. Putensen et al. [13] and Varpula et al. [10] set *P*_high_ below UIP and *P*_low_ above LIP allowing to reach zero flow during the release phase. Li et al. set *P*_low_ to 0 but used *P*–*V* curve to set *T*_low_ to obtain an intrinsic end expiratory pressure (PEEP) of 2 cmH_2_O above LIP. On the other hand, the other two studies [11, 16] set *P*_high_ according to plateau pressure measured during conventional ventilation, with a *P*_low_ of 5 cmH_2_O. Then, *T*_low_ was set to reach 50–75% of peak expiratory flow rate.

About outcomes, all studies reported length of mechanical ventilation. Two studies reported hospital mortality [11, 16], and one studied reported ICU mortality [11].

### Risk of bias and quality of evidence

The results of the quality assessment of included studies are given in Table 2. Two studies have a low bias for random-sequence generation using a computer-based randomization [11, 16]. Three studies have a low risk of bias for allocation concealment as they use sealed envelopes [10, 11, 16]. Li et al. [12] did not clearly define inclusion and exclusion criteria. Due to the nature of the intervention investigated, blindness was not possible for any studies exposing to a high risk of performance bias. None of the studies included mentioned a blindness of outcome assessment.

**Table 2:** Quality assessment of the included studies

	Random-sequence generation	Allocation concealment	Blinding of participants and personnel	Blinding of outcome assessment	Incomplete outcome data	Selective reporting	Other bias
Putensen et al. [13]	?	?	–	?	+	?	–
Varpula et al. [10]	?	+	–	?	+	?	?
Li et al. [12]	?	?	–	?	+	?	?
Zhou et al. [11]	+	+	–	?	+	+	+
Hirshberg et al. [16]	+	+	–	?	+	+	+

+, low risk of bias; –, high risk of bias; ?, unclear risk

### Primary outcome

**Fig. 2:**
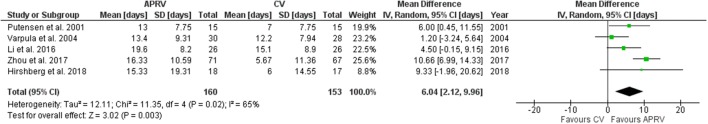
Ventilator-free days at day 28. *APRV* airway pressure release ventilation, *CV* conventional ventilation

Five RCTs including 313 patients reported ventilator-free day at day 28 [10–13, 16]. The overall MD between APRV and conventional ventilation was 6.04 days (95%CI 2.12, 9.96, *p *= 0.003; *I*^2^ = 65%, *p *= 0.02) (Fig. 2). The sensitivity analysis performed excluding two studies [10, 13] confirmed the result (MD 8.03 days [(95%CI 3.42, 12.65, *p *< 0.001; *I*^2^ = 52%, *p *= 0.12]). As the studies included were less than ten, a publication bias analysis was not performed.

### Secondary outcomes

**Fig. 3:**
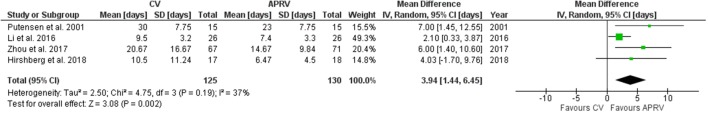
ICU LOS. *APRV* airway pressure release ventilation, *CV* conventional ventilation

**Fig. 4:**
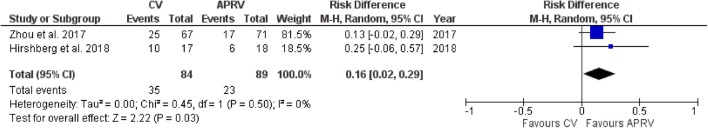
Hospital mortality. *APRV* airway pressure release ventilation, *CV* conventional ventilation

**Fig. 5:**
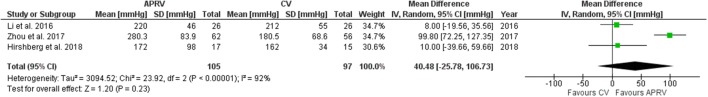
PaO_2_/FiO_2_ at day 3. *APRV* airway pressure release ventilation, *CV* conventional ventilation

**Fig. 6:**
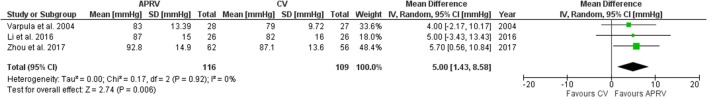
MAP at day 3. *APRV* airway pressure release ventilation, *CV* conventional ventilation

**Fig. 7:**
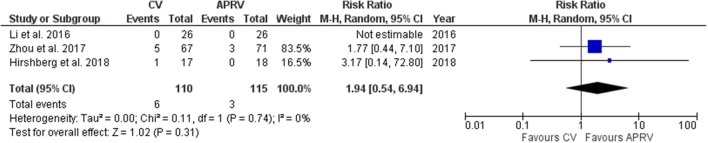
Barotrauma. *APRV* airway pressure release ventilation, *CV* conventional ventilation

Patients treated with APRV had a lower ICU LOS than patients treated with conventional ventilation (MD 3.94 days [95%CI 1.44, 6.45, *p *= 0.002; *I*^2^ = 37%, *p *= 0.19], Fig. 3) and a lower hospital mortality (RD 0.16 [95%CI 0.02, 0.29, *p *= 0.03; *I*^2^ = 0, *p *= 0.5], Fig. 4). PaO_2_/FiO_2_ at day 3 was not different between the two groups (MD 40.48 mmHg [95%CI − 25.78, 106.73, *p *= 0.23; *I*^2^ = 92%, *p *< 0.001] Fig. 5). MAP at day 3 was significantly higher during APRV (MD 5 mmHg [95%CI 1.43, 8.58, *p *= 0.006; *I*^2^ = 0%, *p *= 0.92] Fig. 6). Four studies did not show any differences in vasoactive drugs use and dosage between groups [10–12, 16]. Only one study demonstrated a lower dose of norepinephrine and dobutamine in APRV group [13]. Then, there was no difference regarding the onset of pneumothorax under the two ventilation strategies (RR 1.94 [95%CI 0.54, 6.94, *p *= 0.31; *I*^2^ = 0%, *p *= 0.74] Fig. 7).

All studies reported data about sedation. However, the heterogeneity for reporting this outcome was very high and a quantitative analysis was not possible. Two studies have shown no difference in the dosage of analgo-sedative drugs [10, 16]. On the other hand, two studies demonstrated a lower sedation depth for patients treated with APRV than conventional ventilation and a lower dose of sedative drugs [11, 13]. Finally, Li et al. [12] have recorded more days without sedative drugs in APRV group.

Three studies reported data about spontaneous breathing in APRV patients. Zhou et al. [11] reported that the amount of spontaneous breathing was about 24% of total ventilation at day 1, increasing progressively up to about 26% at day 7. Similarly, Putensen et al. [13] showed that spontaneous breathing accounted for 10% of total ventilation at day 1 and raised up to 35% at day 10. Finally, Hirshberg et al. [16] reported that patients on APRV were able to develop a spontaneous tidal volume of 6.2 ml/kg over a total volume of 8.3 ml/kg at day 1.

ICU mortality was reported only by Zhou et al. [11] with no statistical significance between the two groups (APRV 23.9%, conventional ventilation 37.3%; *p *= 0.088).

## Discussion

This study was the first systematic review and meta-analysis investigating the clinical effects of APRV in adult patients affected by acute hypoxemic respiratory failure compared with conventional ventilation. We demonstrated that APRV was associated with higher ventilator-free days at 28 day, lower ICU LOS and lower hospital mortality, without any major impact on cardiovascular system and risk of barotrauma.

APRV may be considered a general approach of ventilation rather than a single unified ventilatory strategy. The conceptual aim of APRV is to maximize and maintain alveolar recruitment applying the *P*_high_ for the most time of ventilatory cycle and allowing spontaneous breathing. The purpose is to stabilize and rest the open lung reducing the repetitive alveolar collapse and expansion limiting the ventilator-induced lung injury. The rational and the optimal application of APRV has been recently reviewed by Nieman et al. [17] Some evidences suggest that this strategy resulted in better oxygenation and respiratory compliance with less applied pressure than conventional ventilation [11]. Spontaneous breathing allowed during any phase of ventilatory cycle may have several advantages [18]. A better *V*/*Q* match has been demonstrated with a reduction in dead space and intrapulmonary shunt [19] due to the increased aeration in dependent lung regions and the increased lung perfusion [20]. Lower value of pleural pressure during spontaneous breathing may also be responsible for better hemodynamics parameters observed during APRV, with increased venous return, increased preload and consequently increased cardiac output [13]. In our study, although patients treated with APRV had a statistically significant higher value of MAP than patients on conventional ventilation, the difference was not probably clinically significant (MD 5 mmHg; 95%CI 1.43, 8.58, *p *= 0.006) and we can substantially state that APRV has no adverse effect on blood pressure. Although we have considered only MAP in our quantitative analysis, clinical evidences showed that APRV has no negative effects on cardiac index (CI). Varpula et al. [10] showed no difference between groups, while other two studies demonstrated higher values of CI when APRV was used [12, 19]. Then, APRV needs less sedation level than conventional ventilation, which may explain the lower ICU LOS of these patients than whom heavily sedated to allow total controlled ventilation. Finally, another beneficial effect of spontaneous breathing is maintenance of diaphragmatic function.

However, despite the numerous positive aspects of APRV, several concerns have been raised about spontaneous breathing, especially during the most severe cases of ARDS [18]. Spontaneous breathing effort is able to increase trans-pulmonary pressure, and then, increasing the lung stretch, it increases the risk of lung injury [21, 22]. A great variability in tidal volume during APRV has been also demonstrated that may be responsible for the development of larger tidal volume exceeding the protective ventilation. Furthermore, edema may be worse because of increased trans-vascular pressure promoting by negative pleural pressure [23]. Thus, it has been demonstrated that early short term neuromuscular blockade was associated with lower mortality in patients with severe ARDS [24].

Taking this concern in mind, APRV may probably be considered in case of mild/moderate ARDS, when the potential benefits may overcome the potential risks.

This meta-analysis has several limitations. We included a low number of studies, and the overall quality level of them is low. This is due to the lack of blindness and the lack in reporting the management of other potential bias (Table 2). Only two studies have a low risk of bias [11, 16]. Moreover, the heterogeneity for the primary outcome (ventilator-free days at 28 day) was quite high, probably because of the methodological differences between trials. Two studies [10, 19] enrolled patients before the publication of ARDSNetwork trial [3], showing the benefit of low tidal volume ventilation. Thus, for these studies, a higher tidal volume than protective ventilation was permitted. Excluding them in the sensitivity analysis, the heterogeneity decreased without any significant impact on overall effect. Patient’s selection may also explain some heterogeneity. Stratification based on PaO_2_/FiO_2_ was not possible so that patients with different severity of disease may be considered all together. Moreover, the different time of ventilation before application of APRV may affect the outcome. Finally, another aspect to consider is the different initial setting of APRV. Despite it was described for the first time in 1987, a standard setting is not reached yet and some variability in its used is described in the literature. The lower hospital mortality of APRV group than patients on conventional ventilation shown by our study must be interpreted with caution. In fact, only two trials were included in this analysis with an overall population of only 173 patients.

## Conclusion

This study showed a higher number of ventilator-free days at 28 day and a lower hospital mortality in acute hypoxemic patients treated with APRV than conventional ventilation, without any negative hemodynamic impact or higher risk of barotrauma. However, these results need to be interpreted with caution because of the low-quality evidence supporting them and the moderate heterogeneity found. Thus, based on these evidences, it is difficult to draw a clinical message about APRV in this specific setting and other well-designed RCTs need to be conducted to confirm our findings.

## Abbreviations

ALI: acute lung injury
APRV: airway pressure release ventilation
ARDS: acute respiratory distress syndrome
ICU: intensive care unit
LIP: lower inflection points
LOS: length of stay
LTV: low tidal volume
MD: mean differences
PEEP: positive end expiratory pressure
*P*_high_: pressure high
*P*_low_: pressure low
*P*–*V*: pressure–volume curve
RCTs: randomized controlled trials
RD: risk difference
RR: risk ratio
SD: standard deviation
*T*_high_: time high
*T*_low_: time low
UIP: upper inflection points
*V*/*Q*: ventilation/perfusion matching

## References

[1] Bellani G, Laffey JG, Pham T, Fan E, Brochard L, Esteban A (2016). Epidemiology, patterns of care, and mortality for patients with acute respiratory distress syndrome in intensive care units in 50 countries. JAMA.

[2] Fan E, Del Sorbo L, Goligher EC, Hodgson CL, Munshi L, Walkey AJ (2017). An official American Thoracic Society/European Society of Intensive Care Medicine/Society of Critical Care Medicine clinical practice guideline: mechanical ventilation in adult patients with acute respiratory distress syndrome. Am J Respir Crit Care Med.

[3] Brower RG, Matthay MA, Morris A, Schoenfeld D, Thompson BT, Acute Respiratory Distress Syndrome Network (2000). Ventilation with lower tidal volumes as compared with traditional tidal volumes for acute lung injury and the acute respiratory distress syndrome. N Engl J Med.

[4] Stock MC, Downs JB, Frolicher DA (1987). Airway pressure release ventilation. Crit Care Med.

[5] Liberati A, Altman DG, Tetzlaff J, Mulrow C, Gøtzsche PC, Ioannidis JPA (2009). The PRISMA statement for reporting systematic reviews and meta-analyses of studies that evaluate health care interventions: explanation and elaboration. Ann Intern Med.

[6] Higgins J, Green S. Cochrane handbook for systematic reviews of interventions. 5.1.0. 2011. www.handbook.cochrane.org. Accessed Dec 2018.

[7] Wan X, Wang W, Liu J, Tong T (2014). Estimating the sample mean and standard deviation from the sample size, median, range and/or interquartile range. BMC Med Res Methodol.

[8] Higgins JPT, Thompson SG (2002). Quantifying heterogeneity in a meta-analysis. Stat Med.

[9] Egger M, Smith GD, Schneider M, Minder C (1997). Bias in meta-analysis detected by a simple, graphical test. BMJ.

[10] Varpula T, Valta P, Niemi R, Takkunen O, Hynynen M, Pettilä V (2004). Airway pressure release ventilation as a primary ventilatory mode in acute respiratory distress syndrome. Acta Anaesthesiol Scand.

[11] Zhou Y, Jin X, Lv Y, Wang P, Yang Y, Liang G (2017). Early application of airway pressure release ventilation may reduce the duration of mechanical ventilation in acute respiratory distress syndrome. Intensive Care Med.

[12] Li J, Li N, Han G, Pan C, Zhang Y, Shi X (2016). Clinical research about airway pressure release ventilation for moderate to severe acute respiratory distress syndrome. Eur Rev Med Pharmacol Sci.

[13] Putensen C, Zech S, Wrigge H, Zinserling J, Stüber F, Von Spiegel T (2001). Long-term effects of spontaneous breathing during ventilatory support in patients with acute lung injury. Am J Respir Crit Care Med.

[14] Bernard GR, Artigas A, Brigham KL, Carlet J, Falke K, Hudson L (1994). The American-European Consensus Conference on ARDS. Definitions, mechanisms, relevant outcomes, and clinical trial coordination. Am J Respir Crit Care Med.

[15] ARDS Definition Task Force (2012). Acute respiratory distress syndrome: the Berlin definition. JAMA.

[16] Hirshberg EL, Lanspa MJ, Peterson J, Carpenter L, Wilson EL, Brown SM (2018). Randomized feasibility trial of a low tidal volume-airway pressure release ventilation protocol compared with traditional airway pressure release ventilation and volume control ventilation protocols. Crit Care Med.

[17] Nieman GF, Andrews P, Satalin J, Wilcox K, Kollisch-Singule M, Madden M (2018). Acute lung injury: how to stabilize a broken lung. Crit Care.

[18] Yoshida T, Fujino Y, Amato MBP, Kavanagh BP (2017). Fifty years of research in ARDS. Spontaneous breathing during mechanical ventilation. Risks, mechanisms, and management. Am J Respir Crit Care Med.

[19] Putensen C, Mutz NJ, Putensen-Himmer G, Zinserling J (1999). Spontaneous breathing during ventilatory support improves ventilation–perfusion distributions in patients with acute respiratory distress syndrome. Am J Respir Crit Care Med.

[20] Neumann P, Wrigge H, Zinserling J, Hinz J, Maripuu E, Andersson LG (2005). Spontaneous breathing affects the spatial ventilation and perfusion distribution during mechanical ventilatory support. Crit Care Med.

[21] Yoshida T, Uchiyama A, Matsuura N, Mashimo T, Fujino Y (2012). Spontaneous breathing during lung-protective ventilation in an experimental acute lung injury model: high transpulmonary pressure associated with strong spontaneous breathing effort may worsen lung injury. Crit Care Med.

[22] Yoshida T, Uchiyama A, Matsuura N, Mashimo T, Fujino Y (2013). The comparison of spontaneous breathing and muscle paralysis in two different severities of experimental lung injury. Crit Care Med.

[23] Kallet RH, Alonso JA, Luce JM, Matthay MA (1999). Exacerbation of acute pulmonary edema during assisted mechanical ventilation using a low-tidal volume, lung-protective ventilator strategy. Chest.

[24] Papazian L, Forel J-M, Gacouin A, Penot-Ragon C, Perrin G, Loundou A (2010). Neuromuscular blockers in early acute respiratory distress syndrome. N Engl J Med.

